# Phytocannabinoids Biosynthesis in Angiosperms, Fungi, and Liverworts and Their Versatile Role

**DOI:** 10.3390/plants10071307

**Published:** 2021-06-28

**Authors:** Yamshi Arif, Priyanka Singh, Andrzej Bajguz, Shamsul Hayat

**Affiliations:** 1 Department of Botany, Plant Physiology Section, Faculty of Life Sciences, Aligarh Muslim University, Aligarh 202002, India; yamshiarifalig@gmail.com (Y.A.); singh.priyanka8156@gmail.com (P.S.); hayat_68@yahoo.co.in (S.H.); 2 Department of Biology and Plant Ecology, Faculty of Biology, University of Bialystok, Ciolkowskiego 1J, 15-245 Bialystok, Poland

**Keywords:** abiotic stress, cell homeostasis, heterologous host synthetic approach, terpenophenolics

## Abstract

Phytocannabinoids are a structurally diverse class of bioactive naturally occurring compounds found in angiosperms, fungi, and liverworts and produced in several plant organs such as the flower and glandular trichrome of *Cannabis sativa*, the scales in *Rhododendron*, and oil bodies of liverworts such as *Radula* species; they show a diverse role in humans and plants. Moreover, phytocannabinoids are prenylated polyketides, i.e., terpenophenolics, which are derived from isoprenoid and fatty acid precursors. Additionally, targeted productions of active phytocannabinoids have beneficial properties via the genes involved and their expression in a heterologous host. Bioactive compounds show a remarkable non-hallucinogenic biological property that is determined by the variable nature of the side chain and prenyl group defined by the enzymes involved in their biosynthesis. Phytocannabinoids possess therapeutic, antibacterial, and antimicrobial properties; thus, they are used in treating several human diseases. This review gives the latest knowledge on their role in the amelioration of abiotic (heat, cold, and radiation) stress in plants. It also aims to provide synthetic and biotechnological approaches based on combinatorial biochemical and protein engineering to synthesize phytocannabinoids with enhanced properties.

## 1. Introduction

**Figure 1:**
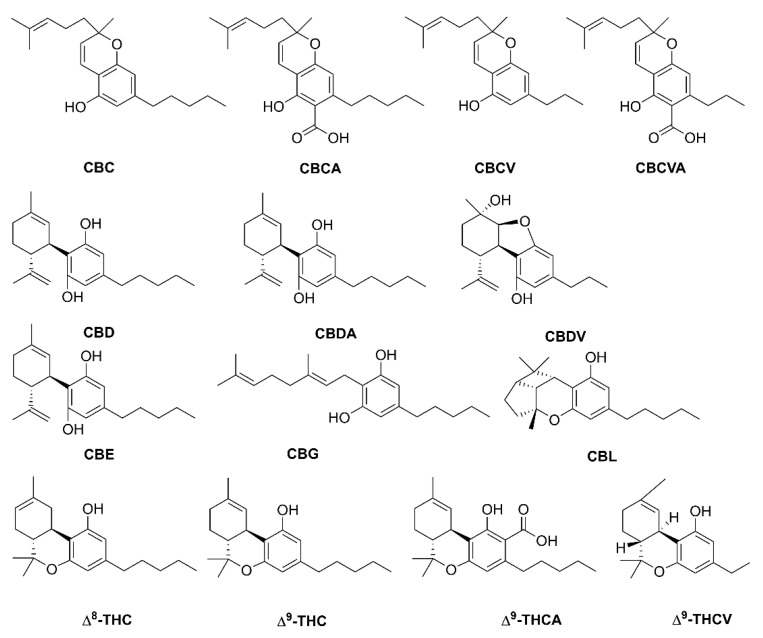
Structure of phytocannabinoids in *Cannabis sativa*. Abbreviations: CBC, cannabichromene; CBCA, cannabichromenic acid; CBCV, cannabichromevarine; CBCVA, cannabichromevarinic acid; CBD, cannabidiol; CBDA, cannabidiolic acid; CBDV, cannabidivarine, CBE, cannabielsoin; CBG, cannabigerol; CBL, cannabicyclol; Δ^8^-THC, Δ^8^-tetrahydrocannabinol; Δ^9^-THC, Δ^9^-tetrahydrocannabinol; Δ^9^-THCA, Δ^9^-tetrahydrocannabinolic acid; Δ^9^-THCV, Δ^9^-tetrahydrocannabivarinic acid.

Phytocannabinoids are meroterpenoids bearing a resorcinol core with an isoprenyl, alkyl, or aralkyl para-positioned side chain, or alkyl group usually containing an odd number of carbon atoms—cannabinoids that have an even number of carbon atoms in the side chain are rare. Phytocannabinoids can be obtained from angiosperms (flowering plants), fungi, and liverworts (Figure 1). The first phytocannabinoid was isolated from the *Cannabis sativa* family Cannabaceae, but it has a long controversial history of its use and abuse [1,2]. From *C. sativa* more than 113 phytocannabinoids were isolated and classified into several groups such as cannabidiols (CBDs), cannabigerols (CBGs), cannabicyclols (CBLs), cannabidiols (CBNDs), cannabinols (CBNs), cannabitriols (CBTs), cannabichromenes (CBCs), (−)-Δ^9^-*trans*-tetrahydrocannabinol (Δ^9^-THC) and miscellaneous cannabinoids [1,3,4,5]. Compounds obtained from *C. sativa* predominately generate alkyl-type phytocannabinoids with a monoterpene isoprenyl and the pentyl side chain [4,6]. In *C. sativa*, CBD, CBG, CBC, cannabichromevarine (CBCV), and Δ^9^-THC are the most abundant cannabinoids in their respective acidic form. The acidic form of the cannabinoid (C22, “pre-cannabinoids”) is the final step of the cannabinoid biosynthetic pathway. Oxidation, decarboxylation, and cyclization lead to the development of modified phytocannabinoid via spontaneous breakdown or conversion product. The conversion mainly occurs due to the poor oxidative stability of phytocannabinoids, especially with the alkyl group. *C. sativa* produces the most common phytocannabinoids. In addition to this, the brains of mammals have receptors that respond to the *C. sativa* cannabinoid, so they were termed as cannabinoid receptor types 1 and 2 (CB_1_R and CB_2_R) and thus participated in the endocannabinoid system [1,3,4,7,8].

**Figure 2:**
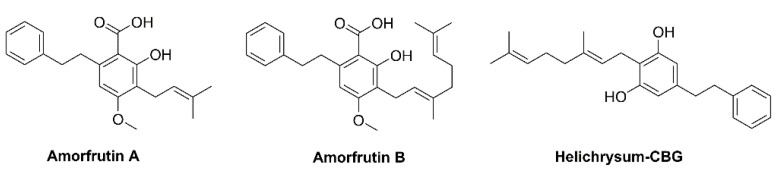
Structure of phytocannabinoids in *Helichrysum* and *Glycyrrhiza* plants.

The Endocannabinoid system in humans and animals revealed that it participates in the regulation of biological functions such as memory, brain system, mood and addiction along with cellular and metabolic processes such as glycolysis, lipolysis, and the energy balance system [7,9]. Other angiosperms such as *Helichrysum umbraculigerum* (Asteraceae) native to South Africa, *Amorpha fruticosa* (Fabaceae), and *Glycyrrhiza foetida* (Fabaceae) contains a bioactive compound bearing a cannabinoid backbone (Figure 2); they are characterized as prenylated bibenzyl derivatives because the aralkyl side chain occurs [1,10].

**Figure 3:**
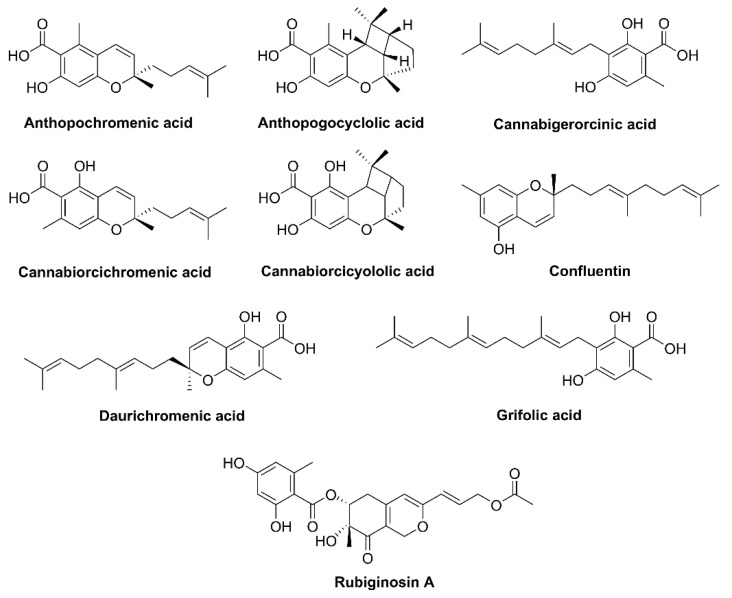
Structure of phytocannabinoids in *Rhododendron* plants.

Many *Rhododendron* species (family Ericaceae) such as *Rh. dauricum* native to Northeastern Asia, *Rh. adamsii* found in Eastern Siberia and Mongolia, *Rh. rubiginosum* var. *rubiginosum* native to Southwest China, and *Rh. anthopogonoides* grown in Southern China, all generate active monoterpenoids that have a cannabinoid backbone. Phytocannabinoids are CBC types with an orcinol or methyl group side chain (Figure 3). *Rh. dauricum* particularly produces cannabinoids bearing sesquiterpene moiety such as daurichromenic acid (DCA), grifolic acid (GFA), confluentin, and rhododaurichromenic acid [11,12,13]. *Rh. adamsii* produces cannabigerorcinic acid, DCA, cannabigerorcinic acid methylase, chromane, and chromene monoterpenoids; *Rh. rubiginosum* produces cannabinoid rubiginosins A–G [14,15]. *Rh. anthopogonoides* contains chromane/chromene derivatives such as cannabiorcicyclolic acid, cannabiorcichromenic acid, anthopogochromenic acid, and anthopogocyclolic acid (Figure 3) [16].

**Figure 4:**
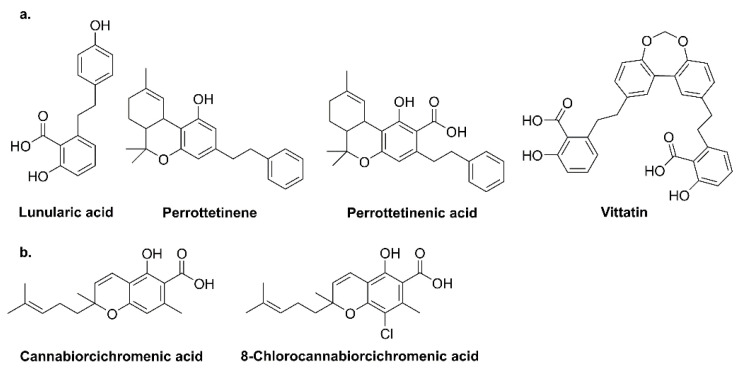
Structure of phytocannabinoids in (**a**) liverworts and (**b**) fungi.

Liverworts, such as *Radula marginata*, *R. perrottetii,* and *R. laxirameae* native to New Zealand, produce active cannabinoids with bibenzyl backbones such as lunularic acid and its dimeric form—vittatin (Figure 4) [17,18,19,20]. Some fungi, e.g., *Albatrellus* (Albatrellaceae, mycorrhizal fungi) species, also produce GFA along with its derivative confluentin, grifolin, and neogrifolin (Figure 4). Additionally, *Cylindrocarpon olidum* generates cannabiorcichromenic acid and halogenated cannabinoid, i.e., 8-chlorocannabiorcichromenic acid (Figure 4) [1,21].

This review focuses on the biosynthesis of different active phytocannabinoids in several cellular compartments of *C. sativa*, *Rhododendron*, and *Radula* species. In this topic framework, the most crucial criterion is the synthetic and biotechnological techniques for the production of phytocannabinoids. The current review highlights the multi-faceted role of different active phytocannabinoids in humans and plants. Interestingly, this review briefly highlights the antimicrobial, antibacterial, and antibiotic properties of phytocannabinoid based on recent papers. Additionally, the role of phytocannabinoids in ameliorating pathogenic attack, and environmental stresses, e.g., cold, heat, and UV radiation, is also briefly assessed.

## 2. Phytocannabinoid Biosynthesis Sites

In *C. sativa*, phytocannabinoids are stored in glandular trichomes, located all over the aerial part of the plant, so root surface and root tissues do not keep phytocannabinoid. Female flowers possess a high density of phytocannabinoid [22,23]. Glandular trichomes have balloon shaped secretory vessicle which store cannabinoid. High temperature or herbivory leads to trichome rupture, which releases the sticky contents on the plant parts with viscous and non-crystallizing properties [24,25]. Higher temperatures increase cannabinoid production. Furthermore, cannabinoid production is raised in the cannabis flower after UV-B exposure. Nevertheless, phytocannabinoids act as a sun shield that absorbs lethal UV radiation [26]. *Rhododendron* genus lepidote consists of small leaves surrounded by glandular scales on both the abaxial and adaxial surfaces. These scales have lipophilic globules that contain major bioactive compounds such as cannabinoids, terpenes; the apoplastic space of the glandular scale also contains cannabinoids such as DCA in the *Rhododendron* [27]. Liverworts have oil bodies that are membrane-bound cellular structures that contain cannabinoids, aromatic oil, and terpenoid (*cis* configuration), mostly sesquiterpenoids and diterpenoids. Oil bodies are odiferous bitter, pungent compounds, which make them biologically active. Furthermore, these possess several ecological advantages such as tolerance from temperature, light, or radiation [28,29].

## 3. Biosynthesis of Phytocannabinoids

This section focuses on detailed events undergone in the production of several phytocannabinoids inside *C. sativa*, *Rhododendron*, and liverworts. Moreover, biosynthesis of phytocannabinoids via biotechnological approaches in a heterologous host and synthetic methods are discussed.

### 3.1. Cannabis sativa

Phytocannabinoids are prenylated polyketides, i.e., terpenophenolic compounds, which are derived from isoprenoid and fatty acid precursors. Phytocannabinoid biosynthesis occurs in different cellular compartments: gland cells cytosol, the plastids, and the extracellular storage cavity. In the cytosol, oxidative cleavage of fatty acid such as palmitic acid yields hexanoic acid; it further synthesizes olivetolic acid (OA). The next step is prenylation of phenolic moiety (the polyketide derivatives, 5-pentenyl resorcinolic acid, and OA) with the terpenoid geranyl pyrophosphate (GPP). This step originates from the methylerythritol-4-phosphate (MEP) pathway in plastids. Cyclization (oxidative) and storage of the final products take place outside the gland cells. Transport proteins and vesicle trafficking participate in mobilizing intermediates across the morphologically highly specialized interface between the gland cells and storage cavity [30,31,32].

**Figure 5:**
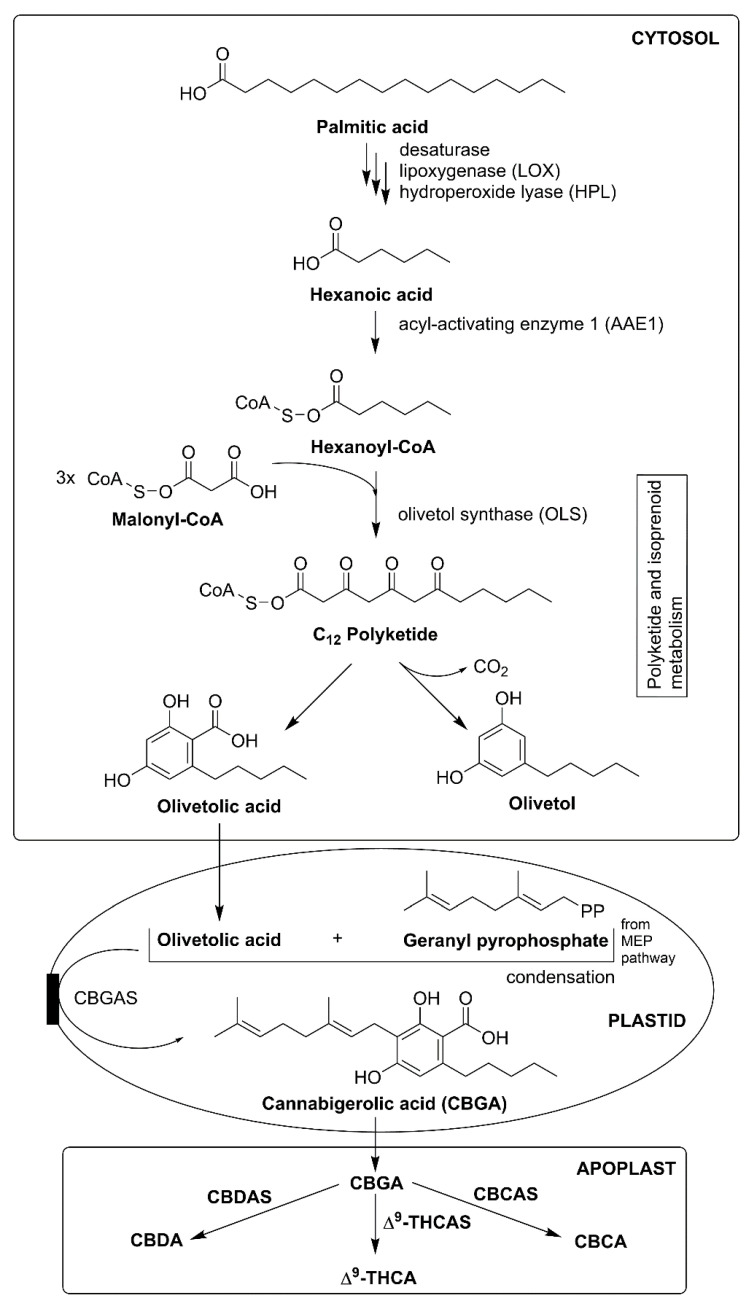
Phytocannabinoids biosynthesis in *Cannabis sativa*. Abbreviations: CBCA, cannabichromenic acid; CBCAS, cannabichromenic acid synthase; CBDA, cannabidiolic acid; CBDAS, cannabidiolic acid synthase; Δ^9^-THCA, Δ^9^-tetrahydrocannabinolic acid; Δ^9^-THCAS, Δ^9^-tetrahydrocannabinolic acid synthase.

In *C. sativa*, phytocannabinoids biosynthesis is divided into three important places: cytosol (for polyketide pathway), plastids (MEP pathway for prenylation), and apoplastic spaces (oxidocyclization and storage) (Figure 5). Inside cytosol, biosynthesis of phytocannabinoids participates in the integration of major steps in polyketide and isoprenoid metabolism. Fatty acids (C18) are sequentially desaturated, peroxygenated, and cleaved into the hexanoic acid (C6) and C12 product via enzyme desaturase, lipoxygenase (LOX), and hydroperoxide lyases, respectively. Hexanoic acid is converted into thioester hexanoyl-CoA; this reaction is catalyzed by acyl-activated enzyme 1 (AAE1). Later, hexanoyl-CoA and malonyl-CoA (C2 donor) together via the action of olivetol synthase (OLS) and olivetolic acid cyclase (OAC) synthesizes OA. Moreover, it was reported that OAC is the dimeric (α+β) barrel protein, it is the first plant enzyme that catalyzes (C2-C7) intramolecular aldol condensation along with carboxylate retention; OAC contains distinctive active-site bearing the pentyl-binding hydrophobic pocket and polyketide binding site, whereas it is devoid of aromatase and thioesterase activities [31,33,34].

Inside plastids, the MEP pathway synthesizes GPP. It prenylates OA, which forms the intermediate branch-point and first cannabinoid which is cannabigerolic acid (CBGA), and this reaction is catalyzed by cannabigerolic acid synthase (CBGAS). CBGA is an essential cannabinoid because it acts as the precursor of several cannabinoids with an alkylic pentyl side chain. In contrast, CBGAS is a transmembrane aromatic prenyltransferase (PT) that transfers plastid signals. Then CBGA is converted into Δ^9^-THCA and cannabidiolic acid (CBDA) with the help of two enzymes which are CBDAS and Δ^9^-tetrahydrocannabinolic acid synthase (Δ^9^-THCAS). This conversion continues by reducing oxygen (O_2_) into hydrogen peroxide (H_2_O_2_) via oxidative cyclization reactions. Additionally, CBDAS and Δ^9^-THCAS are necessary flavoprotein enzymes that are dependent on O_2_ (electron acceptor) [1,30,31].

Another important enzyme, cannabichromenic acid synthase (CBCAS), dependent from FAD and O_2_, takes part in the synthesis of cannabichromenic (CBCA). Additionally, enzymes Δ^9^-THCAS and CBCAS have high sequence similarity at about a 96% nucleotide level. Both remain active inside resin space, which shows that CBCAS participates as an O_2_ dependent flavoprotein that converts CBGA to cannabichromenic acid (CBCA) with H_2_O_2_ as the side product via an oxidocyclization reaction. Active cannabinoid Δ^9^-THCA, CBDA, and CBCA with a pentyl side chain are synthesized in the apoplastic cannabis space. Furthermore, these active phytocannabinoids undergo decarboxylation and spontaneous rearrangement reactions on exposure to heat, radiation, or during storage. Some phytocannabinoid having unknown C1-C4 alkyl side chain are synthesized from acetyl-CoA, propanoyl-CoA, or pentanoyl-CoA [1,5,31,35].

### 3.2. Rhododendron

**Figure 6:**
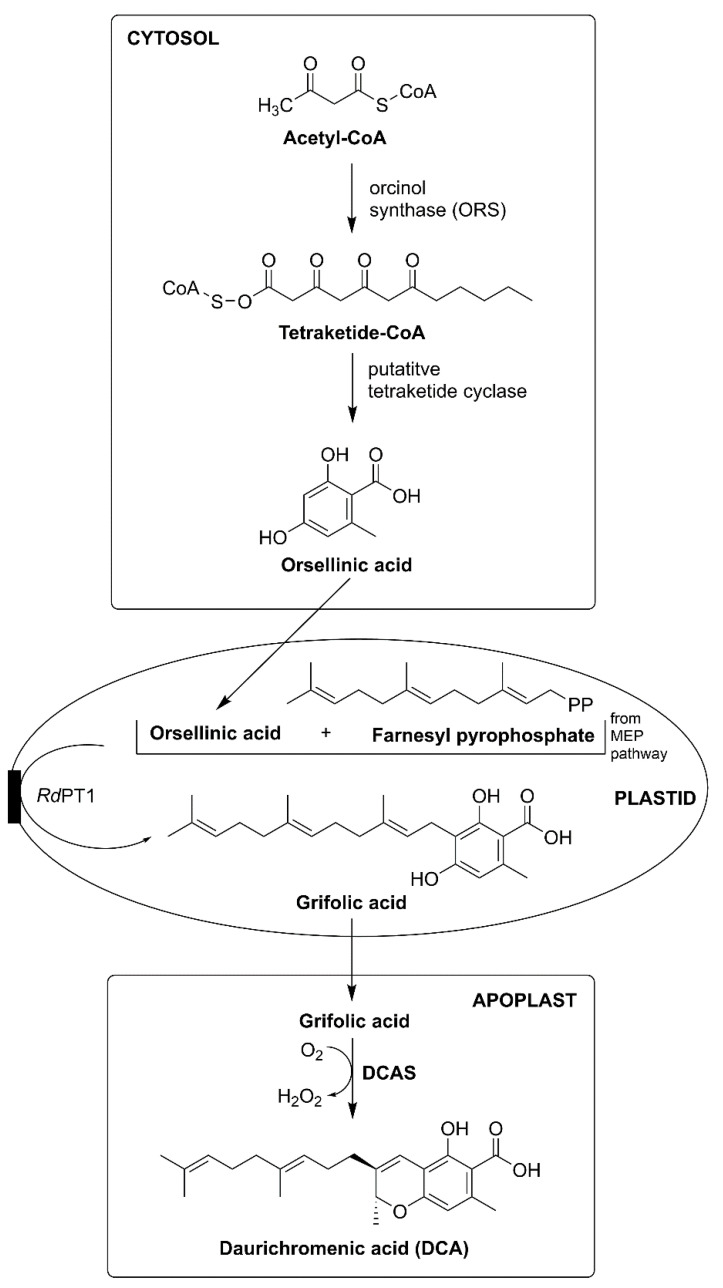
Phytocannabinoids biosynthesis in Rhododendron. Abbreviations: DCAS, daurichromenic acid synthase; MEP, methylerythritol-4-phosphate; PT, prenyltransferase.

DCA and its derivative are produced and stored inside specialized glandular scales in *Rh. dauricum*. DCA utilizes carbon atoms from acetyl-CoA and farnesyl-CoA, with two significant intermediates, i.e., orsellinic acid (OSA) and GFA. DCA biosynthesis in *Rhododendron* is split between the cytosol, plastid, and apoplastic spaces (Figure 6) [11,36].

The biosynthesis of DCA starts in the cytosol with polyketide formation, type III polyketide synthase (PKS) helps in acetyl-CoA chain extension. Then another enzyme, orcinol synthase (ORS), catalyzes orcinol, OSA, triacetic acid, tetracetic acid, lactone and phloroacetophenone, where malonyl-CoA (three units) act as a carbon donor. Furthermore, tetraketide cyclase catalyzes OSA from ORS [36,37]. OA is transported to the plastid via a transporter, which is still unknown. Inside the plastids, the MEP pathway derives farnesyl-CoA. The inhibition of the MEP pathway via clomazone decreases OSA and DCA synthesis. In contrast, inhibition of the mevalonate-dependent pathway via mevastatin led to an increment in OSA and DCA biosynthesis. Moreover, aromatic farnesyltransferase *Rh. dauricum* prenyltransferase (PT) helps in regiospecific farnesylation; this enzyme moderates sequence identity with UbiA aromatic PTs that lie within chloroplasts. Geranyl-CoA and geranylgeranyl-CoA serve as the alternative prenyl donors used by PT, but their activity rate is 13%, and 2.5% of the activity acquired by farnesyl-CoA. GFA is synthesized as the intermediate within the plastids. Then, within apoplastic spaces, an oxidocyclization reaction takes place via DCA synthase (DCAS) forming CBC scaffold; reaction moves forward by H_2_O_2_ release. Like Δ^9^-THCAS and CBDAS, DCAS is active enzymatically outside apoplastic spaces and dependent on O_2_. In *Rh. dauricum*, DCA decarboxylated forms produce confluentin; spontaneous decarboxylation occurs via heat, irradiation, and during storage, similar to the decarboxylation acidic to neutral phytocannabinoids in *C. sativa* trichomes [5,11,37].

Apoplastic spaces serve as storage for many metabolites, essential oils, DCA, and confluentin. Moreover, GFA and DCA act as phytotoxic compounds in *Rh. dauricum* cell culture, as they induce cell death. Similarly, H_2_O_2_ formed as a side product in DCA biosynthesis also increases cell death by enhancing apoptosis-related reactions. However, to overcome cell death, autotoxicity, and cell damage, DCA storage occurs in the apoplast, and H_2_O_2_ is released to participate in the plant-defense system and provide plant immunity. In *Rh. dauricum*, transport proteins and vesicle trafficking mechanisms are still not well understood and remain a valuable and exciting approach for further future investigations [1,2,37].

### 3.3. Liverworts

**Figure 7:**
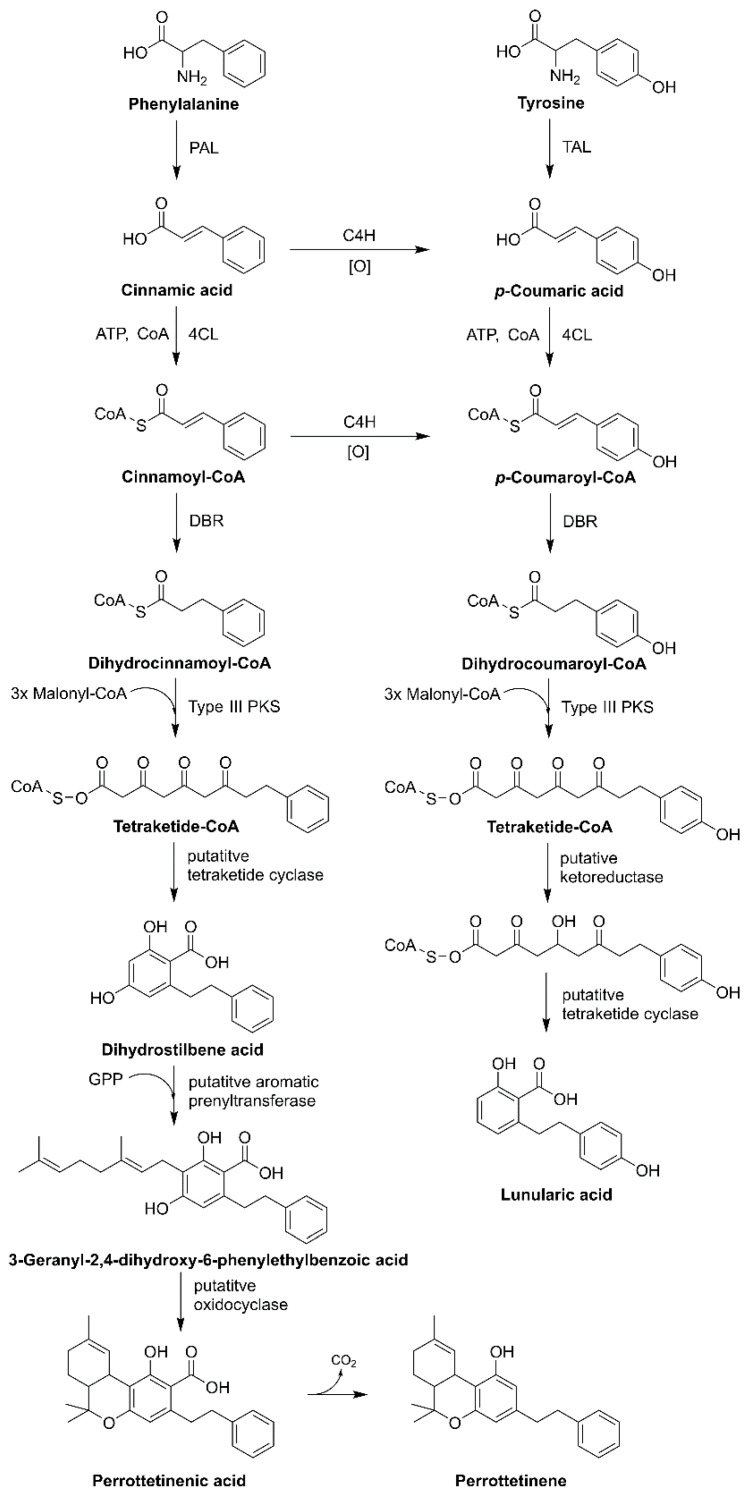
Phytocannabinoids biosynthesis in liverworts. Abbreviations: C4H, cinnamate 4-hydroxylase; 4CL, 4-coumarate:CoA ligase; PAL, phenylalanine ammonia-lyase; TAL, tyrosine ammonia-lyase.

It was reported that *Radula marignata* possesses enzymes for GPP biosynthesis and helps in the biosynthesis of bibenzyl cannabinoid. Moreover, the production of bibenzyl CBGA analog (i.e., lunularic acid, perrottetinenic acid, and perrottetinene) needs precursor stilbene acid or dihydrostilbene acid, which is very rare, and compounds of this type were found in *Hydrangea macrophylla* var. *thunbergii* and liverworts such as *Marchantia polymorpha* and *Convolvulus hystrix* (Figure 7) [1,17,38].

Stilbene acid is synthesized from type III PKS via coumaroyl-CoA or dihydrocoumaroyl-CoA, CoA-activated precursors. Starter molecules are extended by malonyl-CoA with decarboxylation, followed by a condensation reaction, which produces polyketide intermediate, which synthesizes different core structures. Hydrangic acid is the starter molecule that acts as coumaroyl-CoA. It is extended utilizing malonyl-CoA (three units) as a C2 donor to synthesize tetraketide intermediate. These reactions are catalyzed via stilbene synthase (STS)-type PKS enzymes. Ketoreductase (KR) leads to polyketide reduction followed by STS-like C2 to C7 intramolecular aldol condensation, here retention of the carboxylic group produces hydrangic acid. KR is involved in the loss of the C5-hydroxyl group on the aromatic ring structure of hydrangic acid, contrary to the stilbene acid structure. In *R. marginata*, the precursor of stilbene acid or dihydrostilbene is derived from the coumaroyl CoA or cinnamoyl-CoA; type III PKS enzyme helps in chain elongation, later putative tetraketide cyclase or (dihydrostilbene acid cyclase, DHAC) helps in cyclization [1,18,38,39].

The lunularic acid precursor, prelunularic acid is produced by the type III PKS, named bibenzyl synthase (BBS). Moreover, BBS catalyzes the reaction where dihydrocoumaroyl-CoA serves as the starter molecule for the extension utilizing malonyl-CoA (3-units), which serves as the carbon donor. Later, cyclization occurs on reduced polyketide, with a lack of C5-hydroxyl group on the aromatic ring structure. Furthermore, type III PKS plays a crucial role in the bibenzyl cannabinoid and its analog synthesis by also catalyzing the carboxylate retaining reaction mechanism. KR is involved in the cyclization and assists proper ring formation. Thus, after cyclization, lunularic acid is synthesized [40,41,42].

Perrottetinenic acid (PA) is synthesized in *R. marginate* [43]. The transcriptomic approach of liverworts bears the mRNA encoding for type III PKS (responsible for chain elongation), which were later recognized as STS. Furthermore, it also exhibits a 60% homology of the amino acid sequence to stilbene-carboxylate synthase in *Marchantia polymorpha*. Other enzymes, such as double-bond reductase (DBR)—aromatic PT and oxidocyclase (perrottetinenic acid synthase, PAS), are also responsible for the production of PA. DBR catalyzes compounds that are precursors of phenylpropanoid, and generates dihydrocinnamoyl-CoA. The production of bibenzyl phytocannabinoid is dispersed across the liverworts cells in the same way as in cannabis and *Rhododendron*. Therefore, DHAC and DBR reside in the cytosol, PT is localized in the plastids and PAS inside the oil body. Signal peptides act as the crucial indicators for the encoding genes selection [18,39,41,44]. Another bibenzyl *cis*-THC, (−)-*cis*-perrottetinene (*cis*-PET) was isolated from the liverwort *R. perrottetii* [45]. The *cis* configuration in the cyclohexene ring in *cis*-PET is comparable with Δ^9^-*trans*-THC. PET resembles Δ^9^-THC in its 3D shape, and can bind to many of the same cannabinoid receptors (CBRs) as Δ^9^-THC. Interestingly, PET also reduces the level of prostaglandins in the brain—a compound with inflammatory properties that increase in response to Δ^9^-THC and may be responsible for adverse effects [17].

### 3.4. Application of Biotechnological Approaches to Phytocannabinoids Production in Heterologous Hosts

Phytocannabinoids can be generated in different heterologous hosts such as fungi, bacteria, and plants with the help of biotechnological techniques. Several investigations reported that in vitro culture of phytocannabinoids biosynthesis in *C. sativa*, with the help of explants and micropropagation, is a widely used biotechnological approach for phytocannabinoid production. Moreover, apart from *C. sativa*, *Nicotiana benthamiana* also emerged as the favorable heterologous host for the production of phytocannabinoids. It exhibits the production of several proteins and bioactive compounds and has glandular trichomes that help overcome cell death, autotoxicity, and cell damage caused due to intermediates produced during phytocannabinoids biosynthesis. In recent research, it was found that a major biotechnological tool for phytocannabinoids production and inducing genetic modification is established via the micropropagation technique. Additionally, cell suspension culture, hairy root, and adventitious root culture also produce a small quantity of cannabinoids. In *Saccharomyces cerevisiae*, galactose produces phytocannabinoids such as CBG, CBDA, Δ^9^-THCA, and minor phytocannabinoids such as cannabidivarinic acid (CBDVA) and Δ^9^-tetrahydrocannabidivarinic acid (Δ^9^-THCVA) [46,47,48,49,50,51].

### 3.5. Production of Phytocannabinoids through Synthetic Approaches

Phytocannabinoids are terpenophenolic compounds that are produced by polyketide and the MEP pathway. In the heterologous biosynthesis approach, two important pathways that are optimizing and engineering provide abundant precursors for cannabinoid production. Additionally, these pathways are linked via aromatic PT, ubiquitous in plants, animals, bacteria, and fungi [52,53]. Thus, this helps in the production of aromatic metabolites such as coumarin, flavonoid, and phenylpropanoid at different reaction spectra [54]. As discussed, biosynthesis of phytocannabinoid is different in plants, fungi, and liverworts; thus, the recent techniques which emerge out to be beneficial is by using the aromatic PT-based approach to generate novel phytocannabinoids in the heterologous hosts formed on combinational utilization of module over several species [52,53,54].

Similar to *Humulus lupulus*, inside trichomes of *C. sativa*, chalcone isomerases such as proteins (CHILs) are expressed. Additionally, CHILs are polyketide binding proteins, and their co-expression in heterologous cannabinoid production mechanism plays a pivotal role in augmenting biosynthesis. Apart from combinatorial biochemistry techniques, enzyme-based approaches also play a remarkable role in phytocannabinoid production. Furthermore, some non-natural precursors such as pentanoic acid, hexanoic acid, heptanoic acid are incorporated inside the generated cannabinoid. Derivatization of phytocannabinoid diversifies their functionality. Glycosylation of phytocannabinoid reduces cell damage and autotoxicity and increases cell stability and life; in a therapeutic context, glycosylated phytocannabinoid enhances absorption, distribution, metabolism and, excretion (ADME) features. Halogenation of cannabinoids (like DCA) by co-expression of halogenase AscD derived from *Fusarium* exhibits several antifungal, antibacterial, antitumor, antiparasitic properties. Thus, the synthetic biology technique serves as a new insight into the production and designing of phytocannabinoids [55,56,57,58].

## 4. Phytocannabinoid Storage and Maintenance of Cell Homeostasis

The last step of phytocannabinoid biosynthesis and their storage both occur in different cellular compartments like in C. sativa storage organs are resin present inside glandular trichomes, in Rh. dauricum they storage takes place inside apoplast of glandular scale while in R. marginata oil bodies are the storage organs [1]. In *C. sativa*, nuclear magnetic resonance (NMR) based metabolomics of trichomes revealed storage of phytocannabinoids along with terpenes, sugars, organic acid, amino acid, and choline [6]. Sugars, organic acids, and choline, which present in appropriate stoichiometric ratios, give rise to natural deep eutectic solvents (NADES) [59]. NMR-based techniques revealed that constituents for NADES display proton-associated intermolecular interactions, forming aggregates to form larger structures in the liquid phase [60]. NADES is a distinguished solvent bearing this property when the water content is less than 40%; this unique property of NADES incorporates them in the third membrane-less solvent phase inside biological systems [61,62]. Δ^9^-THCA and CBDA support NADES mediated solubilization in trichomes and oil bodies as they are virtually insoluble in water. In *C. sativa* and *Rh. dauricum*, oxidocyclase releases H_2_O_2_. Furthermore, it participates in a plant-defense system and provides plant immunity. NADES stabilizes the activity of oxidocyclases, whereas it also serves as a biological solvent where the biosynthetic catalytic enzyme remains active and functional. NADES plays a crucial role in a plant cell to maintain cell homeostasis. Thus, it plays a chief role in stabilizing phytocannabinoid biosynthesis enzymes, their storage and further induces cellular homeostasis. Recently, cytochrome P450 (CYP) enzymes, CYP79A1, CYP71E1, and P450 oxidoreductase involved in the biosynthesis of the cyanogenic glucoside dhurrin, which was stabilized NADES. Biosynthesis and appropriate storage of cannabinoids in heterologous hosts need to be engineered to reduce autotoxicity and apoptosis [63,64].

## 5. Biotechnology and In Vitro Propagation of Cannabis

Cannabis is an essential plant as it yields phytocannabinoid, which has multiple applications. Cannabis cultivation is regulated in several countries, so alternative biotechnological and in vitro tissue culture approaches require attention. Meanwhile, these approaches are beneficial to preserve cultivars or clones of plants with specific metabolites such as phytocannabinoid. Micropropagation generates genetically stable plants; thus, this method is beneficial for the clonal multiplication of cannabis. Nevertheless, micropropagation of the cannabis plant is performed via adventitious buds and axillary buds located on the nodal segment. Another method that is synthetic seed technology (encapsulation of axillary bud or nodal segment in calcium alginate seeds) has also gained importance in plant clonal propagation. It displays homogenous growth and development of plants and genetic stability even after long storage [65,66].

Cannabis is not a well-known recalcitrant plant for transformation. The plant’s regeneration capacity is low and is completely dependent on plant tissue, age, explants, and a combination of plant growth regulators (PGRs), i.e., indole-3-acetic acid (IAA), indole-3-butyric acids, 1-naphthaleneacetic acid (NAA), 2,4-dichlorophenoxyacetic acid, and kinetin. Moreover, successful transformation is performed via *Agrobacterium tumefaciens*; cells that do not regenerate the shoots are undifferentiated and can be transformed via phosphomannose isomerize and calorimetric assays, which showed efficacious expression of the transgene [67]. Cannabis transformation using shoot tip explants regenerates a high amount of cannabinoid after infection with *Agrobacterium tumefaciens* [68,69]. For the successful regeneration of cannabis, thidiazuron (behavior as cytokinin-like compound) with kinetin (represent cytokinins) has not only the effect on the shoots but also shows a high yielding capacity of phytocannabinoids. Dicamba (3,6-dichloro-2-methoxybenzoic acid) herbicide is also known for inducing the regeneration of shoots from calli. Moreover, a most potent and efficient transformation of cannabis was carried out using 1–2 cm of hypocotyl explants and by supplementation of zeatin and 6-benzylaminopurine [65].

Another important tissue culture technique used for the production of bioactive cannabinoids showing pharmaceutical effects is hairy root culture. In this system, both *Agrobacterium tumefaciens* and *Agrobacterium rhizogenes* are used for transformation to yield cannabinoids by establishing a hairy root system. Meanwhile, the most responsive tissue for infection in this system is hypocotyl, which produces large quantities of hypocotyls or engineered plants for the production of bioactive compounds that are industrially valuable; for example, Δ^9^-THCA is produced by expressing Δ^9^-THCAS. The hairy root technique is obtained by the increased growth rate, independent of a hormone, and has the same metabolic potential as the original plant organ. In the hairy root system, cannabis callus was cultured on a full-strength B5 medium feed with an NAA or IAA displayed increase in the accumulation of phytocannabinoids. Rhizogenesis induction in cannabis undifferentiated cells is crucial, as it can be performed on calli overexpressing some important transcription factors (TFs) and genes which are involved in cannabinoid synthesis. Additionally, cannabis hairy root culture is an efficient method implemented with adsorbents to reduce toxicity problems [65,67].

Cannabis cell suspension culture transformation with the genes involved in the cannabinoid synthesis pathway offers a great opportunity to enhance bioactive phytocannabinoid production possessing pharmacological potential. Additionally, increased production via cannabis cell suspension culture can be obtained through TF. TF have cascade mechanism (that is when the master regulator of cannabinoid synthesis is identified; there it can be expressed inducibly or constitutively inside cell suspension culture). Thus, TF constitutes potent and efficient tool involved in plant metabolic engineering [65].

TF, belonging to the MYB family, was involved in cannabinoid production and tolerant oxidative stress. TF expression in an inducible manner confers toxicity tolerance caused due to the accumulation of phytocannabinoids during the growth of transformed cells. Genetic engineering and plant cell suspension culture were elicited to induce the production of specific cannabinoids. Additionally, in cannabis suspension cells, induction with biotic and abiotic elicitors did not cause an increment in phytocannabinoid synthesis. Non-essential elements, such as silicon, play a key role in mitigating biotic and abiotic stress. Additionally, silicon has a stimulatory effect on the production of cannabis secondary metabolites like cannabinoids. Cyclodextrins are cyclic oligosaccharides bearing five or more than five α-D-glucopyranose residues; these form an inclusion complex structure with lipophilic compounds like cannabinoids. In plant cell suspension culture, cyclodextrins are used for the production of non-polar compounds such as stilbenes and other cannabinoids. Moreover, cyclodextrins improve cannabinoid and other metabolite solubilities in an aqueous environment. Cyclodextrins possess a similar structure to alkyl-derived oligosaccharide released from the plant cell wall during a fungal infection; thus they act as elicitors for the production of secondary metabolites. More investigations are needed on the effect of cyclodextrins on the synthesis of non-polar cannabinoids in cannabis suspension cultures [65,70].

## 6. Phytocannabinoids and Their Derivative Role and Bioactivity in Humans and Animals

Phytocannabinoids are used as medicines since antiquity. They possess antimicrobial, antibacterial, and antifungal activity and serve as remarkable antibiotics (Table 1). Additionally, cannabinoids show little antifungal power because fungi can metabolize cannabinoids except a few, such as *Phomopsis ganjae*. An increasing number of developing countries are relaxing their legislation around phytocannabinoids and cannabis-derived products; legalized cannabis-derived product industries are increasing worldwide. They are likely to constitute a projected US$ 57 billion market by the year 2027 [71].

### 6.1. Cannabinoid Receptors in Humans and Their Role

CB_1_R (cannabinoid receptor type 1, first cloned in 1990) mRNA is highly expressed in the brain. It is also found in the heart, lung, ovary, adrenal gland, thymus, testes, prostate, tonsils, bone marrow, and uterus. CB_1_R is also widely distributed inside non-neuronal tissues and inside various cells, tissues and also co-exist with other CBRs [86,87]. CB_1_R is found inside the brain and displays high protein density and expression inside brain parts such as the cerebellar molecular layer, substantia nigra pars reticulate, globus pallidus external and internal, olfactory bulb, olfactory nucleus (anterior), hippocampus and layers II–III, Va and VI of the cerebral cortex. Additionally, in humans, the highest CB_1_R level is found inside the cingulated gyrus, motor cortices and frontal secondary somatosensory. CB_1_R is found in moderate levels inside the hypothalamus and ventral striatum and low levels in the brainstem; other respiratory control centers lack CB_1_R. Moreover, this is the main reason CB_1_R has a low effect on respiratory and cardiovascular activities [88]. Another important receptor, CB_2_R, has high density inside immune cells and tissues, whereas a low amount is expressed inside the brain. Inside the brain, most of the presynaptic CBRs are observed as CB_1_R, whereas it was displayed that presynaptic inhibitory CB_2_R in GABAergic terminals of the hippocampus [86,87]. Phytocannabinoid applies its strong physio-psychotropic and psychotogenic actions along with the modulation of fast synaptic transmission in the brain showing its action on receptor CB_1_R, present in the synaptic region. Additionally, fast synaptic activity encloses signaling via GABA and glutamate (activate ionotropic receptors). However, CB_1_R contributes to psychoactivity and neurophysiology along with phytocannabinoid [89]. CB_2_R is found inside pyramidal cells (II/III) of the medial prefrontal cortex; when it activates, it causes IP3-dependent opening of calcium-activated chloride channels and ultimately resulted in the inactivation of neuronal firing [90,91].

G-protein-coupled receptors (GPR), such as GPR18 and GPR55, serve as potent phytocannabinoid receptors. GPR18 is a “deorphanized” receptor present in cells and tissues of the thymus, spleen, small intestine, lymph nodes, leukocytes, and gametes [92,93,94,95]. Phytocannabinoids (e.g., Δ^9^-THC) are agonists to GPR18. It plays a potent role in signalizing toxin-sensitive G-protein, Akt, PI3K, and p42/44 mitogen-activated protein kinase [96,97]. Additionally, cannabinoid (e.g., Δ^9^-THC) induces β-arrestin recruitment in the cells which are transfected with GPR18 [86,95]. GPR55 is the important “deorphanized” metabotropic receptor that interacts with several active phytocannabinoids; it is present in the central nervous system (CNS), adipose tissues, adrenal gland, immune cells, small intestine, osteoblasts and osteoclast [98,99]. GPR55 displays a low sequence with both CB_1_R and CB_2_R (about 15%); GPRs interact with Gα12 or Gα13, which results in activation of several pathways such as Rho-associated protein kinase, Ras homolog gene family member A (RhoA), p38, MAPK/ERK pathways [91,100]. Some phytocannabinoids potently activate GPR5. Moreover, GPR55 is the most complex CBR. The same ligand acts as an agonist, antagonist, or neutral; it also possesses the allosteric modulator site [86,87]. Phytocannabinoids serve as weak and partial agonists on the GPR55, but via the help of some cannabinoids (CBD, cannabidivarine, CBDV), they inhibit lysophosphatidylinositol linked GPR55 activation. Thus, GPR55, along with cannabinoids, can participate in curing cancer cells, obesity, inflammation, neuropathic pain, osteoporosis, and neuromodulation. Transient receptor potential (TRP) superfamily channels have 27 polymodal sensor cation channels classified into six broad types in humans [101]. Among these six families, four groups interact with cannabinoids and other derivatives. Thus, these participate in boosting the immune system, with analgesia (pain reliever), and nociception (processing of sensory nervous system); they also modulate inflammations and pain sensations [102,103,104].

Phytocannabinoids exerts strong therapeutic potential in humans assessed by the meta-analysis of several clinical trials; it possesses a strong effect on health conditions such as nausea, vomiting, insomnia (sleeping disorder), depression, anxiety, paraplegia (paralyzes in a lower limb due to spinal cord injury), psychosis (emotional metal disorder), appetite stimulation in several syndromes [86]. Additionally, cannabinoids play a significant role in ailments of human diseases and syndromes such as Tourette syndrome (nervous system disorder such as continuous movements), AIDS, and treating intraocular pressure of the eye in glaucoma [105]. The endocannabinoid system (internal lipid retrograde neurotransmitters which bind on CBR) along with phytocannabinoid is involved in modulating pharmacological, physiological, biological, and cognitive processes [106]. Due to the limited space, in this review, we only name diseases and ailments cured by phytocannabinoids that are liver encephalopathy, hepatic disease, asthma, respiratory tract changes, bronchospasm, bone remodeling and metabolism, osteoarthritis, and osteoporosis. Phytocannabinoids may also be used to treat diseases related to CNS such as brain trauma, stroke, brain aging, neuroinflammation, and neurodegradation. Additionally, they have served as an anti-solid depressant to cure patients with suicidal tendencies; apart from all this, they are a strong pain reliever and immunomodulator. Phytocannabinoids are also given to pregnant women to avoid miscarriage and preimplantation embryo development [86,105,107].

Phytocannabinoids have several medicinal properties in curing diseases and disorders and assist as an important template for chemists to form novel and synthetic medicine [108]. Several investigations display the potential of synthetic cannabinoids in human ailments. Δ^9^-THC and CBD together or alone form synthetic cannabinoids such as nabiximols, Sativex^®^, nabilone, dronabinol, levonantradol, and other synthetic Δ^9^-THC-analogs. These synthetic cannabinoids are used to cure cancer pain, neuropathic pain, and spasticity caused by sclerosis [75,105].

Phytocannabinoids have diverse therapeutic potential, but they have several adverse risks, mainly due to fewer trials. Certainly, there is a necessity to support research and investigation on phytocannabinoid legally and ethically; globally, the legalization of cannabinoids encounters several controversies by society, researchers, and health practitioners. Many countries such as Canada and the US also support phytocannabinoid production for medical use. Colorado’s state is employing private support from the medical cannabinoid (marijuana) industry to endorse future research on phytocannabinoids and cannabis [108,109].

### 6.2. Bioactivity of Phytocannabinoids

Phytocannabinoids are of great clinical use in humans and mammals, but the plant sources do not accumulate at high levels and are also regarded as endangered species. This enhances the demand for the biosynthesis of phytocannabinoids via biotechnological approaches to understand their bioactivity and role in medication. The prerequisite and establishment for such biotechnological approaches should be proper and deep knowledge of the pathways and mechanisms involved in phytocannabinoid production in *C. sativa*, liverworts and *Rhododendron* species [1,2,78,105].

#### 6.2.1. Neutral Cannabinoids

In humans, phytocannabinoids play a potent role in signal transduction. They show crucial interaction with the G-protein coupled cannabinoid receptors (GPCRs such as CB_1_R and CB_2_R), peroxisome proliferator-activated receptor γ (PPARγ), and TRP ion channel. CB_1_R is the most abundant GPCR located in CNS, whereas CB_2_R is found in immune cells and tissues. Thus, cannabinoids play a crucial role in signaling, immune, and CNS proper functioning [72,74]. In humans, Δ^9^-THC displays pleiotropic effects such as analgesic (relieve pain), relaxation, pain tolerance, and dysphoria (anxiety disorder); thus this reveals Δ^9^-THC displays agonistic effects by the activation of CB_1_R of β-arrestin 2 recruitment and signaling; also Δ^9^-THC is the essential psychoactive (alters mood, behavior, feeling and thoughts by affecting CNS) bioactive constituent [73,104]. Δ^9^-THC is found in the drug dronabinol (Marinol^®^), and the constituent of sesame oil plays potent antiemetic (prevents vomiting) for cancer patients receiving chemotherapy. Additionally, it also stimulates appetite in a patient with AIDS (acquired immunodeficiency syndrome); chronic administration of doses increases weight and appetite. Δ^9^-THC is also given to patients with insomnia and depression as it improves sleep. In humans, Δ^8^-THC decreases intraocular pressure (fluid pressure inside the eye) and exhibits antiglaucoma activity [76]. Phytocannabinoid CBG possesses non-psychotic activity that has a lower affinity for both CB_1_R and CB_2_R, several times lower than Δ^9^-THC. CBG has remarkable activity towards ligand-gated cation channels of superfamily TRP. CBG serves as an agonist of TRP type ankyrin 1 (TRPA1) and TRP type vanilloid 1 (TRPV1), whereas it acts as an antagonistic of TRP type melastatin 8 (TRPM8). Δ^9^-tetrahydrocannabivarinic acid (Δ^9^-THCV) serves as a non-psychoactive that is antagonistic to CB_1_R. In particular, Δ^9^-THCV potentially acts against obesity-linked glucose intolerance. In humans, it acts as the potential phytocannabinoid for treating metabolic disorders, hepatosteatosis syndrome, and obesity. It also helps recover fasting plasma glucose and pancreatic β cell functioning. Additionally, Δ^9^-THCV reduces adiponectin and apolipoprotein [75].

CBD plays a potent pharmacological role in humans by affecting CNS and some peripheral portions, as it is highly antagonistic to CB_1_R and CB_2_R. Additionally, CBD also acts as an essential allosteric modulator of receptor μ-opioid (pain reliever), thus used to relieve pain [75]. In humans, doses in the range of 10 to 700 mg CBD are not toxic and are delivered to patients in the form of Epidiolex^®^ with epilepsy treatment-resistant, interlinked with CDKL5 deficiency disorder and many other disorders and syndromes [80]. CBD does not act as a psychoactive compound and can be delivered in patients receiving pharmacotherapy. Furthermore, CBD reduces Δ^9^-THC-elicited psychotic disorders and decreases the deleterious effect of Δ^9^-THC on memory which is hippocampus-dependent. CBD acts as the potential cannabinoid to cure obesity, convulsive disorder, and rheumatoid arthritis. Furthermore, CBD also possesses antipsychotic, antinausea, and antianxiety properties [79].

CBC acts as the non-psychotropic which does not interact with CB_1_R and CB_2_R [5,78]. Additionally, CBC inhibits endocannabinoid inactivation and further activates TRPA1, which helps recover intestinal inflammation and has several protective roles [110,111]. Additionally, CBC also possesses anti-inflammatory activity for curing lipopolysaccharide-enhanced edema. In particular, till 2021, no CBC study involves humans [112].

#### 6.2.2. Cannabinoid Acid

In *C. sativa*, CBDA, CBCA, CBDA, CBGA, and Δ^9^-THCA belong to the cannabinoid acid group, which does not possess cannabimimetic or psychotropic bioactivity [5]. Continuous decarboxylation of Δ^9^-THCA leads to a low level of Δ^9^-THC [113]. Δ^9^-THCA has more affinity than Δ^9^-THC and binds to PPARγ, whereas it has a low affinity towards CB_1_R and CB_2_R. Additionally, Δ^9^-THCA possesses the neuroprotective property [77]. Phytocannabinoids of cannabis and their derivatives hamper the level of tumor necrosis factor α after stimulating lipopolysaccharide in culture supernatants of some macrophages. Additionally, phytocannabinoids, i.e., cannabinoid acid, also display their effect on phospholipase activity specific to phosphatidylcholine; thus, this effects signaling. Cannabinoid acids, e.g., CBDA, possess antihyperalgesic (i.e., reduce abnormally enhance pain) and anti-inflammatory properties [114]. In mice, cannabinoid acid (i.e., CBDA, and Δ^9^-THCA) treatment increased antihyperalgesic and anti-inflammatory properties with inflammations induced by carrageenan. Additionally, in mice, oral administration of cannabinoids increases the antihyperalgesic property before carrageenan. In intestinal segments of house musk shrews, CBDA enhances tissue reduction in the resting state with the help of non-neuronal-associated pathways or pathways that are independent or are not associated with CB_1_R or CB_2_R [82,115].

#### 6.2.3. Bibenzyl Cannabinoids

Analogs of bioactive bibenzyl (aralkyl) compound such as CBG from few liverworts have a phenethyl side chain; so bibenzyl CBG displays reduced affinity to cannabinoids receptors [116]. Additionally, it has an affinity to TRPA1 and TRP 1-4, which are ionotropic receptors, and shows a strong association to TRPM8 [10]. Bibenzyl phytocannabinoids belonging to the class amofrutin were extracted from *Glycyrrhiza foetida* and *Amorpha fruticosa*. They possess anti-inflammatory properties and are a strong activator of PPARγ [84]. Bibenzyl cannabinoid perrottetinene produced by *Radula* species shows a structural resemblance to cannabis Δ^9^-THC, distinguishing it by having an aromatic side chain instead of a pentyl. In mammals, perrottetinene acts as the psychoactive compound that is agonistic to CB_1_R. Furthermore, it enhances behavioral tetrads of analgesia, catalepsy, hypolocomotion, and hypothermia. Interestingly, enantiomers produced in liverworts are the major hallmark of their bioactivity and metabolic responses [28].

#### 6.2.4. Rhododendron Cannabinoids

*Rhododendron* produces phytocannabinoids in the form of prenylated orcinoids. They are generally classified in CBC or CBL type as they have chromane or chromene scaffold. Prenylated orcinoids and their derivatives play a potent role in building and boosting up the immune system in mammals and humans. *Rhododendron* cannabinoids possess anticancer property; hence can be recommended to a cancer patient. It also has antithrombotic activity. Furthermore, it also displays anti-inflammatory, antimicrobial, and antipsychotic properties and has very low toxicity in humans when administered [5,37,83,117]. Other essential phytocannabinoids of the *Rhododendron* species, DCA and rhododaurichromenic acids, serve as the potent bioactive compounds in curing HIV as they possess anti-HIV bioactivities. It was found that when DCA was administered in infected H9 cells, it showed a half-maximal effective concentration (EC_50_) of 15 nM, much lower than the azidothymidine (a drug used to cure HIV), which has EC_50_ of 44 nM. Moreover, DCA with potent anti-HIV bioactivity has a therapeutic index (TI) of 3701 and EC_50_ of 5.67 ng mL^−1^ [81]. Moreover, rhododaurichromanic acid possesses anti-HIV properties with TI of 92 and EC_50_ of 370 ng mL^−1^. Active phytocannabinoids from *Rh. anthopogonoides* such as cannabiorcichromenic acid (CBC-type), anthopogocyclolic acid, and anthopogochromenic acid, which are CBL-type possess antiallergic activity as they inhibit the release of histamine [5,16,117].

## 7. Antibacterial and Antimicrobial Property of Phytocannabinoids

Cannabis fibers are used in textile industries as they are rich in active phytocannabinoids, which provide high biomass in less time. Hemp fibers, also called bast fibers, are rich in Δ^9^-THC, cellulose, lignin, and other metabolites, so they are used in industries, animal bedding, and used as a substitute for glass fibers. Hemp fibers also possess high antibacterial properties because of the presence of phytocannabinoids, so they are used as antibacterial finishing agents and in surgical industries. Recently it was found that cannabis powder contains a high amount of cannabinoid content which possesses antibacterial properties against *Escherichia coli*. Interestingly, phytocannabinoids have high pharmacological, antibacterial, and antimicrobial activities [65,78,105,118,119].

Cannabis extract contains antibacterial property against *Bacillus subtilis* and *Staphylococcus aureus,* which are Gram-positive bacteria; also against Gram-negative bacteria *E. coli*, *Pseudomonas aeruginosa*. In contrast, it displays no activity against fungal infections caused by *Aspergillus niger* and *Candida albicans*. Phytocannabinoids, e.g., Δ^9^-THC, CBG, CBN, CBD, and CBC, possess antibiotic properties against methicillin-resistant *Staphylococcus aureus*. CBD and Δ^9^-THC display bactericidal activity against streptococci and staphylococci. GFA and DCA also show antimicrobial properties against Gram-positive bacteria. Moreover, the prenyl group shows structural similarities to bioactive monoterpenes [58,65,85,108,120].

## 8. Phytocannabinoids in Stress Tolerance

Phytocannabinoids have a diverse role in humans and also exhibit antimicrobial, antibacterial, and antibiotic activity. Apart from this, they also have some other biological beneficial properties in mitigating biotic and abiotic stress in the plant. Cannabis trichomes possess phytocannabinoid in large quantities. High temperatures or herbivory cause trichome rupture and the release of the phytocannabinoid content, which protects the plant from desiccation and high-temperature stress. Therefore, they enhance plant tolerance to heat stress [2,79]. It was also reported that phytocannabinoid production was enhanced in cannabis flowers after UV-B-induced stress. Thus, phytocannabinoids serve as a sun shield against destructive UV-B radiation [26]. Liverworts oil bodies possess phytocannabinoid, so they are not damaged by fungi and bacteria, insect larvae and adults, slugs, snails, and small mammals. Significantly, phytocannabinoids inside oil bodies provide tolerance against several abiotic stresses such as cold temperature, heat, excessive light, and UV radiation [26,29,58].

Additionally, phytocannabinoids provide resilience to desiccation. Liverworts are unable to produce abscisic acid (ABA); besides this, they have phytocannabinoid, i.e., lunularic acid that shows the same activity as ABA, which is a plant hormone. There is a lack of knowledge about the role of phytocannabinoids in biotic and abiotic stress mitigation. Thus, more in-depth studies are still required to understand the mechanism involved in stress tolerance via phytocannabinoids. However, studies must understand their role in other plants’ stress amelioration [1].

## 9. Phytocannabinoid Foe

Why is cannabis cultivation illegal in many countries and what are the disadvantages associated with phytocannabinoid consumption? Cannabis is the third most popular substance consumed after alcohol and tobacco. The USA and New Zealand are at the top rates (42%) of consuming it; however, it is the most illicit drug used globally. Its consumption is escalating among teenagers, and they are consuming it in the form of hashish and marijuana [86,121,122]. It was reported that almost everyone who tried cannabis (marijuana or hashish) becomes a habitual abuser and dependent on cannabis; thus, this enhanced the cannabis use disorder [123,124]. Cannabis elicits a plethora of biological, metabolic, and physical responses. Nevertheless, everything depends on its utilization and what dose it is consumed in (what, when, and how). Most commonly used cannabis marijuana is prepared by drying leaves and flowering buds of the cannabis plant. Moreover, hashish which is eventually stronger than marijuana, is the concentrated resin from the female cannabis plant and is directly consumed via chewing and smoking. Kief is a trichomes mass obtained from flowers and leaves, which is used to make hashish. Additionally, hashish contains a mixture of several phytocannabinoids and terpenes [125]. Smoking or dabbing marijuana is the most common way in the consumption of cannabinoids. Phytocannabinoid delays the onset effect by half to two hour circulating level; circulating level is greater when they are smoked or intravenously consumed. In particular, the delay time in the onset of psychobiological effect is due to the biosynthesis of bioactive phytocannabinoid metabolite or induction in endocannabinoid availability or to reach receptors to show its effect. Cannabis consumption produces two kinds of effects: short-term (acute) and long-term (chronic) [86]. In humans, acute consumption of cannabis effect is classified into two groups: (i) physiological effect, which displays therapeutic potential, and (ii) recreation effects which show the effect on brain identical to psychotic state (psychotomimetic) [126,127]. Acute physiological effects include relaxation, hyperlocomotion, resting of heart, and increase in thirst, appetite, and food palatability. Acute psychotomimetic effects encompass enhanced sociability, euphoria (sense of great happiness), relaxation, hallucinations, delusions, conceptual disorganization, paranoia, social withdrawal, blurred vision, and decreased attention [128,129]. Furthermore, it also leads to anxiety, panic attacks, and dysphoria; all of these effects are similar to schizophrenia. Long-term (chronic) consumption of cannabis is related to addiction risk which depends on age, gender, lifestyle, frequency of intake, dosage, and genetic makeup [122]. Long-term cannabis consumption increases various alterations inside the human body (mainly heart, brain, and nervous system) and develops the serious risk of several disorders such as schizophrenia, neuropsychiatric syndrome, cerebellar infarction, vasoconstriction, and several fatal cardiovascular problems. Additionally, cannabis consumption has a detrimental effect on metabolic and biological processes; but these disorders are low if consumption of alcohol is low; apart from this, cannabis consumption confers useful effects on the risk of cardiometabolic factors such as insulin sensitivity, glycemia, lipid amount. Nevertheless, it remains to be demonstrated whether long-term cannabis consumption in humans functions as an activator or deactivator of CBRs [79,105,108,109,122,126].

## 10. Conclusions and Future Prospects

**Figure 8:**
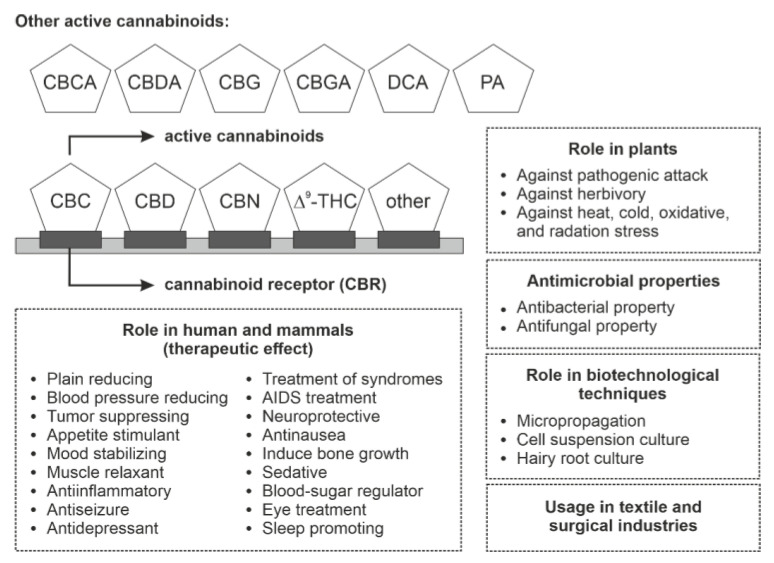
Selective role of active phytocannabinoids in humans, mammals, plants, biotechnology, and industries. Abbreviations: CBC, cannabichromene; CBCA, cannabichromenic acid; CBD, cannabidiol; CBDA, cannabidiolic acid; CBG, cannabigerol; CBGA, cannabigerolic acid; CBN, cannabinol; CBR, cannabinoid receptor; DCA, daurichromenic acid; PA, perrottetinenic acid; Δ^9^-THC, Δ^9^-tetrahydrocannabinol.

Phytocannabinoids are bioactive naturally occurring terpenoids that were earlier thought to be exclusive to *C. sativa* but have now also been produced in *Rhododendron* species, some legumes, the liverwort genus *Radula*, and some fungi. Bioactive phytocannabinoids show remarkable non-hallucinogenic biological properties determined by the variable nature of the side chain and prenyl group defined by the enzymes involved in their biosynthesis. The present review focused on genes and enzymes involved in biosynthesis across several plant species such as cannabis, *Rhododendron* and liverworts were discussed here. Moreover, these species are used in the combinatorial fashion to construct a required new bioactive phytocannabinoid structure established on integrating peculiar prenyl moieties, side chains, and unique cyclized core structures. Meanwhile, phytocannabinoids biosynthesis involves a large collection of enzymes that can potently develop bioactive phytocannabinoids via genetic engineering. This review gives a better understanding of the diverse role of phytocannabinoids in humans, plants, microbiology, and biotechnology (Figure 8). In particular, active phytocannabinoids play a crucial role in treating several diseases in humans. They possess antibacterial and antimicrobial properties in several industries. Recent studies also appraise phytocannabinoids role in cold, heat, and radiation stress tolerance. Phytocannabinoids also protect the plant from pathogens and herbivory. Moreover, the review also focuses on the foe of improper use of phytocannabinoids and why it is banned in many countries.

The hypothesis is that phytocannabinoids have versatile use and are beneficial for humans and plants if appropriately used. Further investigations are needed on genes and enzymes involved in the biosynthetic pathway in different plant species. Comparative study between type III PKS from cannabis and liverwort might explain their specificity to particular molecular structure and insight of genetic and protein engineering. Semi-synthetic techniques and chemical modification integration can be used for the biosynthesis of different types of cannabinoids—it also modulates their bioactivity and bioavailability. The availability of naturally occurring cannabinoids provides an insight into scrutinizing their role in plant stress tolerance, including individual and combinatorial exogenous effects. Lastly, quantitative chemical profiling can also give a deep knowledge of the occurrence and importance of NADES and their other possible role in plants and their role in reducing autotoxicity.

## Figures and Tables

**Table 1:** Bioactivity and effect of different phytocannabinoid in animals and humans.

Phytocannabinoids	Bioactivity in Animals and Human	References
Δ^9^-THC	pleiotropic effects such as analgesic, muscle relaxation, and pain tolerance	[1]
	increases weight and appetite; improves sleep and depression; alters mood, behavior, feeling, and thoughts	[72,73]
	cures neuropathic pain, spasticity, dysphoria, and anxiety disorders	[74,75]
	antiemetic activity, i.e., prevents vomiting in cancer patient during chemotherapy	[76]
	displays antiglaucoma activity and reduces intraocular pressure	[76]
Δ^9^-THCA	neuroprotective and antitumor activity	[77]
Δ^9^-THCV	non-psychoactive effect; cures obesity	[75]
	effective against metabolic disorders, pancreatic disease, and hepatosteatosis syndrome	[1]
CBC	non-psychotropic and anti-inflammatory activity	[77,78]
CBD	cures memory loss, obesity, convulsive disorder, and rheumatoid arthritis	[79]
	cures epilepsy; exhibits antipsychotic, antinausea, and antianxiety activity	[80]
CBDA	anti-inflammatory and antihyperalgesia effect	[81,82]
CBG	non-psychotic activity	[75]
CBC, CBL, and DCA	cures HIV and cancer; possess immune boosting activity	[83]
CBG and amofrutin	anti-inflammatory activity	[84]
CBL	anti-inflammatory, anti-microbial, antipsychotic, and antiallergic activity	[5]
GFA and DCA	anti-microbial activity	[85]
PET	increases analgesia, catalepsy, hypolocomotion and hypothermia	[28]

## Data Availability

Not applicable.

## Abbreviations

AAE1	acyl-activated enzyme 1
ABA	abscisic acid
ADME	absorption, distribution, metabolism, excretion
BBS	bibenzyl synthase
C4H	cinnamate 4-hydroxylase
4CL	4-coumarate:CoA ligase
CBR	cannabinoid receptor
CB_1_R	cannabinoid receptor type 1
CB_2_R	cannabinoid receptor type 2
CBC	cannabichromene
CBCA	cannabichromenic acid
CBCAS	cannabichromenic acid synthase
CBCV	cannabichromevarine
CBCVA	cannabichromevarinic acid
CBD	cannabidiol
CBDA	cannabidiolic acid
CBDAS	cannabidiolic acid synthase
CBDV	cannabidivarine
CBDVA	cannabidivarinic acid
CBE	cannabielsoin
CBG	cannabigerol
CBGA	cannabigerolic acid
CBGAS	cannabigerolic acid synthase
CBL	cannabicyclol
CBN	cannabinol
CBND	cannabidiol
CBT	cannabitriol
CHIL	chalcone isomerases-like protein
CNS	central nervous system
CYP	cytochrome P450
DBR	double-bond reductase
DCA	daurichromenic acid
DCAS	daurichromenic acid synthase
DHAC	dihydrostilbene acid cyclase
Dicamba	3,6-dichloro-2-methoxybenzoic acid
EC_50_	effective concentration
GFA	grifolic acid
GPP	geranyl pyrophosphate
GPR	G-protein-coupled receptor
GPCR	G-protein-coupled cannabinoid receptor
IAA	indole-3-acetic acid
KR	ketoreductase
LOX	lipoxygenase
MEP	methylerythritol-4-phosphate
NAA	1-naphthaleneacetic acid
NADES	natural deep eutectic solvent
OA	olivetolic acid
OAC	olivetolic acid cyclase
OLS	olivetol synthase
ORS	orcinol synthase
OSA	orsellinic acid
PA	perrottetinenic acid
PAL	phenylalanine ammonia-lyase
PAS	perrottetinenic acid synthase
PET	perrottetinene
PGR	plant growth regulator
PKS	polyketide synthase
PPARγ	peroxisome proliferator-activated receptor γ
PT	prenyltransferase
STS	stilbene synthase
TAL	tyrosine ammonia-lyase
TF	transcription factor
TI	therapeutic index
TRP	transient receptor potential
TRPA1	TRP type ankyrin 1
TRPM8	TRP type melastanin 8
TRPV1	TRP type vanilloid 1
Δ^8^-THC	Δ^8^-tetrahydrocannabinol
Δ^9^-THC	Δ^9^-tetrahydrocannabinol
Δ^9^-THCA	Δ^9^-tetrahydrocannabinolic acid
Δ^9^-THCAS	Δ^9^-tetrahydrocannabinolic acid synthase
Δ^9^-THCV	Δ^9^-tetrahydrocannabivarinic acid
Δ^9^-THCVA	Δ^9^-tetrahydrocannabidivarinic acid
